# A needle tip CCEA microfluidic device based on enhanced Dean flow for cell washing

**DOI:** 10.1038/s41378-021-00311-9

**Published:** 2021-10-15

**Authors:** Xin Shi, Wei Tan, Yuwen Lu, Wenfeng Cao, Guorui Zhu

**Affiliations:** 1 School of Chemical Engineering and Technology, Tianjin University, Tianjin, 300350 China; 2 Tianjin Tumor Hospital, Tianjin Medical University, Tianjin, 300070 China

**Keywords:** Chemistry, Nanoscale devices

## Abstract

Particle/cell washing is an essential technique in biological and clinical manipulations. Herein, we propose a novel circular contraction–expansion array (CCEA) microdevice. It can be directly connected to a needle tip without connection tubes. Its small size and centrosymmetric structure are beneficial to low sample consumption, high connection stability, and a wide application range. Computational fluid dynamics (CFD) simulation results show that the CCEA structure can produce a stronger Dean flow and lead to faster particle/cell focusing than the circle structure and CEA structure with the same length. Experimentally, an optimal flow rate ratio of 1:3 and an optimal total flow rate of 120 μL/min were found to ensure a stable fluid distribution. Under these conditions, rapid focusing of 10–20 μm particles with high efficiencies was achieved. Compared with a normal CEA device using tubes, the particle loss rate could be reduced from 64 to 7% when washing 500 μL of a rare sample. Cell suspensions with concentrations from 3 × 10^5^/mL to 1 × 10^3^/mL were tested. The high cell collection efficiency (>85% for three cell lines) and stable waste removal efficiency (>80%) reflected the universality of the CCEA microfluidic device. After the washing, the cell activities of H1299 cells and MCF-7 cells were calculated to be 93.8 and 97.5%, respectively. This needle-tip CCEA microfluidic device showed potential in basic medical research and clinical diagnosis.

## Introduction

Cell washing is a basic biotreatment technology and is widely used in cell identification, culture, and analysis^1–3^. It transfers cells from an original fluid to a new fluid, removing metabolic waste, cell debris, or other unwanted solutes. During cardiomyocyte proliferation, it is used to replenish nutrients^4^. In hemolysis assays of antimalarial drugs, it is used to collect washed human erythrocytes^5^. In flow cytometry, it is used to reduce background interference^6^. The most common method for cell washing is centrifugation. Although the reliability and efficiency of centrifugation are high, evidence shows that high shear rates and centrifugal forces can potentially result in cell damage^7,8^. Some shear-sensitive microalgae can be damaged by residence in the pellet and the g-force applied^9^. The surface properties of staphylococcus can also be altered by centrifugation^10^. Centrifugation is also inefficient in that 27–30% of the cells are lost during the removal process^11,12^. In addition, it is discontinuous and time-consuming.

Compared with centrifugation, microfluidic techniques can manipulate cells and beads in a continuous, sample-saving, and low-stress way^13,14^. The basis of microfluidic cell washing is the precise control of particles and fluids. In inertial microfluidics, particle manipulation can be achieved with a small apparatus and simple operation^15,16^. Gossett et al.^17^ proposed a co-flow system in a straight channel for particle transfer based on the inertial lift force *F*_L_. Particles migrate across laminar streams and enter a new solution without significant disturbance of the interface. Zhou et al.^18^ built a blood–buffer system and enriched leukocytes (which migrate faster than red blood cells) from undiluted whole blood. Dean flow—the secondary flow induced in curving channels—is a well-known inertial effect due to the momentum mismatch of fluid parcels at different cross sections of a channel as these parcels pass around a curve^19^. The particles in Dean flow are driven by the Dean drag force *F*_D_; this force can enhance the lateral migration of particles and alter inertial focusing equilibrium positions. Many excellent works have been produced by researchers that provide guidance on the structure design and operation setting. Sun et al.^20^ reported a double spiral microfluidic device for tumor cell separation from diluted whole blood. The radius of the structure was 9 mm. Hou et al.^21^ described a co-flow microfluidic device to isolate bacteria from whole blood in a spiral channel. With a total length of ~10 cm, the large cells were focused in a band that occupied half the width of the channel. Yin et al.^22^ utilized an eight-loop spiral microchannel device to separate culture-expanded mesenchymal stem cells. Particles with different diameters were focused at a particular flow rate. Johnston et al.^23^ demonstrated a 5-loop spiral microchannel for manipulating particles. The radius of the device was only 3 mm, and different particles were focused at different positions. Bhagat et al.^24^ described a passive microfluidic device with a five-loop spiral microchannel geometry for particle separation. The focused particles were always close to the inner wall with limited lateral migration. In addition to the use of curvature, the introduction of disturbance obstacles into straight channels induces convective secondary flow^25^. Typically, the contraction–expansion array (CEA) structure is widely used by many researchers. Wu et al.^26^ presented a symmetrical CEA microchannel for continuous particle and blood cell separation. The large and small particles were focused on the centerline and near the sidewall, respectively. Based on similar CEA structures, many researchers have focused and separated particles and cells^27–29^. Lee et al.^30^ reported an asymmetrical CEA microfluidic device with sheath flow. The particles initially introduced at the expansion side were entrained in the direction of the counterrotating vortex by the Dean flow at each entrance of the contraction region, which resulted in the migration of particles toward the straight side. Only small particles migrated toward the straight side, while large particles focused close to the expansion side. In addition, Shen et al.^31^ designed a spiral microchannel with ordered micro-obstacles for continuous particle separation. The micro-obstacles could accelerate the secondary flow, which enhanced the particle focusing in time and space. Gou et al.^32^ presented a spiral channel with periodic expansion structures on the outer wall. The large particles were focused on the centerline, and the small particles were focused at two positions close to sidewalls. On the basis of particle focusing, a microfluidic technique has the potential to be modified for cell washing, provided that particles can migrate from the sample to the sheath fluid in a stable co-flow system.

The structures of microchannels also affect the connection stability and sample consumption. For noncentrosymmetric straight CEA and serpentine channels, when a needle is perpendicular to the channel, a cantilever structure is formed by the channel and the needle^33^. This cantilever structure subjects the needle to large bending stress, which reduces the connection stability. When the needle is parallel to the channel, the length of the channel reduces the connection stability. Yun et al.^34^ presented a mechanical cell lysis chip. The device was ~20 mm and required a Luer adapter to connect to the syringe. When this microfluidic device is directly connected to the needle instead of the syringe, the needle is easily bent due to insufficient stiffness. Song et al.^35^ presented a microfluidic method for size-based cell sorting. It was obvious that the device deformed due to the length. In contrast, central symmetry could efficiently improve the stability of the device. Pauli et al.^36^ developed a centrosymmetric lab-in-a-syringe (LIS) for the immunosensing of biomarkers. Xiang et al.^37^ proposed a centrosymmetric syringe flow stabilizer for hand-powered, precise, continuous-flow microfluidic sample injection. A summary of the literature is shown in Table 1.

**Table 1:** Summary of the literature for particle focusing under Dean flow

Author (year)	Structure	Device size	Particle/cell size	Efficiency	Suitability for needle tip
Sun^20^ (2012)	Double spiral	Radius: 9 mm	5 μm	99.66%	Moderate
			15 μm	92.75%	
Hou^21^ (2015)	Spiral	Radius: >15 mm	RBCs	N/A	Moderate
Kuntaegowdanahalli^51^ (2009)	Spiral	Radius: >10 mm	10/15/20 μm	~90%	Moderate
Yin^22^ (2018)	Spiral	Radius: 12 mm	17–21 μm	N/A	Low
Johnston^23^ (2014)	Spiral	Radius: ~3 mm	1.0 μm	~25%	Low
			2.1 μm	<87%	
			3.2 μm	<93%	
Bhagat^24^ (2008)	Spiral	Radius: 4.4 mm	7.32 μm	~100%	Moderate
Bhagat^24^ (2011)	CEA	Length: >15 mm	MCF-7 cells	~80%	Low
Lee^30^ (2013)	CEA	Length: >20 mm	Cancer cells	99.1%	Low
			Blood cells	88.9%	
Zhang^52^ (2013)	CEA	Length: 31 mm	4.8/9.9 μm	N/A	Low
Wu^26^ (2016)	CEA	Length: 45 mm	5.5 μm	92.8%	Low
			9.9 μm	98.3%	
			RBCs	99.8%	
			WBCs	89.7%	
Shen^33^ (2017)	Spiral-obstacles	Radius: >8.5 mm	7.3 μm	90.9%	Moderate
			9.9 μm	98.6%	
			15.5 μm	99.8%	
Gou^32^ (2020)	Spiral-CEA	Radius: >6.5 mm	MCF-7 cells	93.5%	High
			HeLa cells	89.5%	
			A549 cells	88.6%	
This paper	CCEA	Radius: 3 mm	5 μm	57.3%	High
			10 μm	82.6%	
			15 μm	~100%	
			20 μm	~100%	
			H1299 cells	90.6–94.3%	
			MCF-7 cells	93.0%	
			U-2932 cells	85.0%	

Although many inertial microfluidic devices have shown their potential, there are still many challenges to the rapid cell washing of rare samples. First, it is necessary to further optimize the size of the microfluidic device. A short channel length and small size are beneficial to miniaturization and integration. In addition, the distance between the particle focusing position and sample solution should be as large as possible. Considering the nonuniformity of the cell size, the greater the distance is, the higher the washing purity. Finally, directly connecting the washing device to the needle tip is of great significance. Removing tubes can efficiently reduce dead volume. Take a tube with a radius of 0.75 mm and a length of 200 mm as an example. After processing, the sample remaining in the tube reaches 0.4 mL. If the sample is only 1–2 mL, such a loss may be unacceptable. A direct connection can also improve clinical applicability. It is common for operators to acquire samples with needles. If they can wash cells immediately with the initial needle instead of changing to another tube, the operational difficulty, time, and risk of sample contamination can be reduced. The critical parameters for judging whether a microchannel can be used on the needle tip are the central symmetry and device size. A centrosymmetric structure and a small size can make the device more stable during connection and application. The size refers to those of commonly used syringe filters, whose radii are 2, 6.5, 12.5, and 16.5 mm when the volumes of the sample are <1, 1–10, 10–100, and >100 ml, respectively. Thus, we confirm that the radius of the microfluidic device is expected to be less than 6.5 mm when handling rare samples.

In this paper, we propose a circular contraction–expansion array (CCEA) microfluidic device to achieve cell washing on a needle tip. This CCEA structure combines curve structure and CEA structure to strengthen the Dean flow. In this way, rapid particle focusing can be realized after only one loop. Its small size (the radius is only 3 mm) and centrosymmetry allow it to be directly connected with a needle tip, which may reduce sample consumption and simplify operations. The fluid distributions were demonstrated under different flow rates and flow rate ratios. The lateral focusing performances and collection efficiencies (the ratio of target cells collected at the target outlet to both outlets) of particles of different sizes were analyzed. Based on particle investigations, this CCEA microfluidic device was applied to washing three cancer cell lines. The cell collection efficiency and waste removal efficiency were measured by flow cytometry. The cell viability and proliferation after the experiment were observed and recorded. Such a needle-tip device can reduce sample waste and shows advantages in high-precision microfluidic technology.

## Materials and methods

### Ethics statement

All experiments were performed in accordance with the relevant guidelines set by the National Health Commission of the People’s Republic of China and approved by the ethics committee at Tianjin Medical University (Tianjin, China). Informed consent was obtained from human participants in this study.

### Preparation of samples

For particle suspensions, polystyrene (PS) particles of 5, 10, 15, and 20 μm (2.5 wt%, BaseLine Chromtech Research Centre, China) were diluted in phosphate-buffered saline (PBS). Their concentrations were 1 × 10^6^/mL, 1 × 10^5^/mL, 1 × 10^5^/mL, and 1 × 10^5^/mL, respectively. Under such concentrations, particles were monodispersed. To prevent particle aggregation, the surfactant Tween 20 (Solarbio Life Sciences, Beijing) was added to the suspensions at 0.02 w/v%. For cell samples, three human carcinoma cell lines, H1299, MCF-7, and U-2932 (all provided by Tianjin Tumor Hospital, Tianjin, China), were used. The concentrations of H1299 cells were set to 3 × 10^5^/mL, 1 × 10^4^/mL, and 1 × 10^3^/mL. The concentrations of MCF-7 cells and U-2932 cells were 1 × 10^4^ and 1 × 10^5^/mL, respectively. Cells were mixed with fluorescent particles (BaseLine Chromtech Research Centre, China) in PBS. The concentration of exosomes was determined according to the previous work of other researchers. Xu et al.^38^ showed that an ExoPCD chip can efficiently capture tumor-derived exosomes. The range of exosome concentrations varied from 7.61 × 10^4^ to 7.61 × 10^8^/mL. Qian et al.^39^ presented a simple platform for rapid exosome concentration and in situ detection of exosomal microRNA. The exosome concentrations used in the experiments were 5 × 10^6^/mL and 1 × 10^7^ mL. Zhao et al.^40^ successfully detected exosomes for ovarian cancer at a concentration of 7.5 × 10^5^/mL. Vaidyanathan et al.’s nanoshearing technique could specifically detect exosomes from breast cancer patients at a concentration of 2.8 × 10^6^/mL^41^. Thus, the concentration of exosomes in our work was set to 3 × 10^5^/mL. Thus, the ratios of cells to insoluble wastes were ~1:1, 1:30, and 1:300.

### Device design and fabrication

**Fig. 1:**
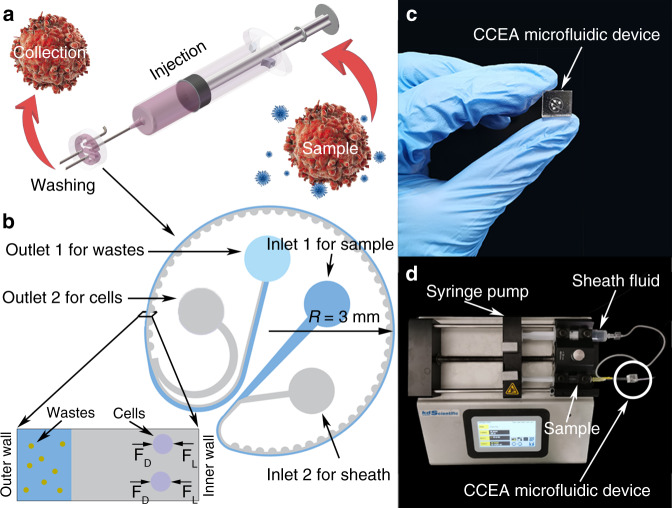
Schematic of cell washing using a CCEA microfluidic device. **a** The main steps of cell washing using a CCEA microfluidic device. **b** The structure of the CCEA microfluidic device. The device consisted of two inlets, two outlets, and 50 repeated contraction–expansion elements. The sample and sheath fluid were injected into the device from inlet 1 and inlet 2, respectively. After the washing, the unwanted wastes and the target cells were collected at outlet 1 and outlet 2, respectively. **c** Photograph of the CCEA microfluidic device. The radius of the device was only 3 mm. **d** Photograph to illustrate the connection configuration.

The CCEA microfluidic device had two inlets (Inlet 1 for sample and Inlet 2 for sheath fluid) and two outlets (Outlet 1 for the target particles and Outlet 2 for the waste fluid), as shown in Fig. 1. The main structure of the device was a circle channel with 50 repeated contraction–expansion elements. The radius of this circle was only 3 mm. The cross section of the main channel was rectangular (100 μm in width). The dimension of the concentration–expansion elements was a semicircle with a radius of 100 μm. The uniform depth of the channel was 40 μm. The CCEA microfluidic device was fabricated by soft lithography techniques using polydimethylsiloxane (PDMS)^42,43^.

### Separation setup and image analysis

All fluids were transferred to syringes and then injected into the CCEA microfluidic device using syringe pumps (Legato 100, KD Scientific, America). All experiments were performed on both needles and glass slides. For the experiments on glass slides, the device was placed onto an inverted microscope (IX73, Olympus, Japan) for observation. Brightfield images were captured by a high-speed camera (Fastcam Mini AX100, Photron, Japan), and fluorescent images were captured by a CCD camera (WY-600D, VIYEE, China). The images were postprocessed and analyzed using ImageJ software (National Institute of Health, Maryland).

### Flow cytometry analysis

To determine the cell collection efficiency and waste removal efficiency, samples at the inlet and outlets were collected and analyzed using a flow cytometer (FACSAria III, BD Biosciences, USA). The H1299 cells were stained with DAPI (Solarbio Life Sciences, China), while the 500 nm particles contained fluorescent dye (excitation wavelength 488 nm, emission wavelength 518 nm). A 375 nm laser was used to excite DAPI fluorescence, which was detected at 450 ± 20 nm. A 488 nm laser was used to excite particle fluorescence, which was detected at 530 ± 30 nm (same as FITC). The flow cytometer measurement was considered complete when the total number of analyzed cells reached 10,000. The data were analyzed with the software FlowJo (TreeStar, San Carlos, CA, USA).

## Results and discussion

### Comparison with the circle structure and CEA structure

**Fig. 2:**
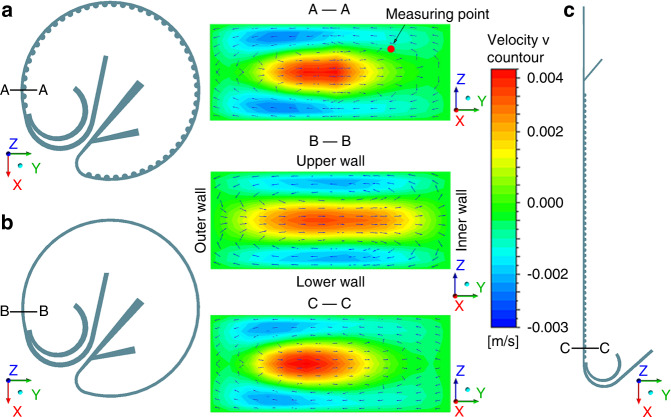
Computational fluid dynamics simulations of different structures. **a** The velocity *u* in the CCEA structure. **b** The velocity *u* in the circle structure. **c** The velocity *u* in the CEA structure.

CFD simulation (ANSYS Fluent 17.0) was used to investigate the velocity distributions in our CCEA structure, the circle structure, and the normal CEA structure. The total flow rate was set to 120 μL/min, consistent with that used in the following experiments. With the same inlet condition and the same cross-sectional area, the velocity distribution on the X-axis of the three structures was basically the same. The contour of the velocity *u* (the velocity on the Y-axis) is shown in Fig. 2. In all three cases, the fluid parcels in the channel center flowed toward the inner wall and then flowed back close to the upper and lower walls. It was obvious that the CCEA structure can produce a much stronger Dean flow. Furthermore, we compared the velocity *u* at the measurement point (10 μm to the upper wall and 20 μm to the inner wall). The velocity *u* in our CCEA structure was 11.7 × 10^−3^ m/s, as shown in Fig. 2a. However, the values were only 0.014 × 10^−3^ m/s and 3.4 × 10^−3^ m/s in the circle structure (Fig. 2b) and the CEA structure (Fig. 2c), respectively. The enhanced Dean flow could accelerate the lateral migration of particles to equilibrium positions and reduce the required channel length (*L*_f_).

The inertial migration of particles in our CCEA structure is dominated by the competition of the inertial lift force *F*_L_ and the Dean drag force *F*_D_. The inertial lift force *F*_L_ comprises the shear gradient lift force and the wall lift force, inducing the lateral migration of particles toward equilibrium positions between the channel centerline and channel walls. This can be expressed as:^44^

1 $$F_L \propto \rho _sU_{\max }^2a^4/D_{\mathop{\rm h}\nolimits} ^2$$

where *ρ*_s_ is the density of the sample, *U*_max_ is the maximum flow velocity, *a* is the particle diameter, and *D*_h_ = 2*wh*/(*w* + *h*) is the hydraulic diameter, with *w* and *h* being the width and height of the straight channel, respectively. The Dean drag force *F*_D_ is due to the difference between the particle velocity and fluid velocity in the cross-sectional plane. It can be calculated by the Stokes drag law:^45^

2 $$F_{\mathop{\rm D}\nolimits} = 3\pi \eta _0a\left( {v_{\mathop{\rm f}\nolimits} - v_{\mathop{\rm p}\nolimits} } \right)$$

where *v*_f_ and *v*_p_ are the lateral velocities of the fluid elements and particles in a cross-sectional plane, respectively.

**Fig. 3:**
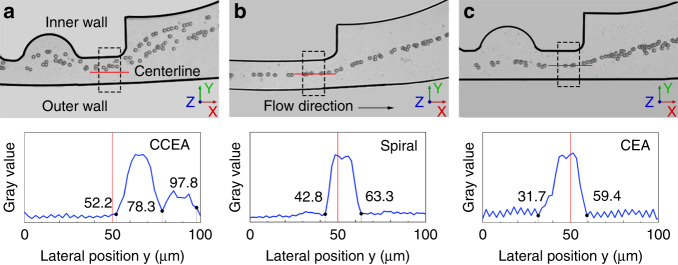
Particle focusing performance in different structures. **a** Composite image from experimental observation of 15 μm particles in the CCEA structure. **b** Composite image from experimental observation of 15 μm particles in the circle structure. **c** Composite image from experimental observation of 15 μm particles in the CEA structure.

The lateral focusing performance was validated using 15 μm PS particles in the three structures: the CCEA, circle, and CEA. (Fig. 3). The flow rates of the sample and sheath fluid were 90 and 30 μL/min, respectively. The particle distributions were observed and analyzed at the end of the main channel. In the CCEA structure, the 15 μm particles initially close to the outer wall were focused on two lines. This means that the inertial lift force *F*_L_ and the Dean drag force *F*_D_ were balanced at two positions on the Y-axis. Both equilibrium positions were close to the inner wall, which showed the significant contribution of *F*_D_ to lateral migration. However, the particles were focused on a single line at the centerline in the circle structure and the CEA structure. The focusing position was similar to that in a straight channel, which showed that the *F*_L_ dominated the lateral migration with negligible influence by the *F*_D_. Therefore, the CCEA structure could generate a larger *F*_D_ for particles than the circle and CEA structures. Based on the larger *F*_D_, the migration distance of the 15 μm particles increased toward the inner wall in such a short channel length. As a comparison, in the circle or the CEA structure, the migration distance was approximately half of the channel width.

### Flow rate and flow rate ratio

**Fig. 4:**
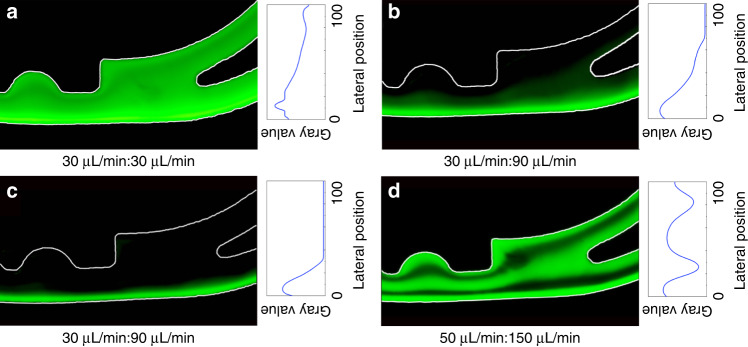
Fluorescence images and gray value analysis of the fluid distributions at the outlets. **a** The flow rates of the sample and sheath fluid were 30 and 30 μL/min. **b** The flow rates of the sample and sheath fluid were 30 and 60 μL/min. **c** The flow rates of the sample and sheath fluid were 30 and 90 μL/min. **d** The flow rates of the sample and sheath fluid were 50 and 150 μL/min. Under the optimal total flow rate of 30 μL/min: 90 μL/min, the sample flowed in a narrow width with little dispersion.

For a clear presentation of the fluid distribution and the interface, fluorescein 2Na (Solarbio) was dissolved in DI water at a concentration of 50 μg/mL. The sample (with fluorescein sodium) and the sheath fluid were injected from inlet 1 and inlet 2, respectively. The flow rate ratio (defined as the ratio of the volumetric flow rate of the fluid at inlets 1 and 2) was adjusted based on a flow rate of 30 μL/min for the sample. The fluorescent imaging area was located at the end of the CCEA channel. The Reynolds number, *Re*, is defined as the ratio of the inertial force to the viscous force:

3 $$Re = \frac{{\rho D_{\mathop{\rm h}\nolimits} V}}{\eta } = \frac{{2\rho Q}}{{\eta \left( {w + h} \right)}}$$

where *D*_h_ = 2*wh*/(*w* + *h*) is the hydraulic diameter, with *w* and *h* being the width and height of the contraction elements; *V* is the maximum fluid velocity in the contraction elements; *η*_0_ is the viscosity of the fluid; and *Q* is the total flow rate. In Fig. 4a, the flow trajectory of the sample is the green part, where the shade indicates the concentration. The area between the sample and the inner wall is the sheath fluid. When the flow rate ratio was 1:1 (Re≈29), the sample layer was too wide at the front of the channel, which led to excessive dispersion at the end. By increasing the flow rate ratio to 1:2 (Re≈43) and 1:3 (Re≈57), as shown in Fig. 4b, c, respectively, the dispersion of the sample layer gradually decreased. It flowed through the whole channel in a steady narrow width. The fluid flowing out from outlet 1 was a mixture of the sample and the sheath fluid, while the fluid flowing out from outlet 2 was only sheath fluid. When the flow rate of the sample was increased to 50 μL/min and the flow rate ratio was 1:3 (Re≈95), as shown in Fig. 4d, the sample layer was no longer close to the outer wall but flowed out from both outlets. This occurred because the higher total flow rate strengthened the Dean flow in the cross section. Thus, the optimal total flow rate of 120 μL/min and flow rate ratio of 1:3 were used in further experiments. Under these conditions, the fluid collected from outlet 2 was only sheath fluid, so this has the potential to be used for particle/cell washing.

### Particle focusing

**Fig. 5:**
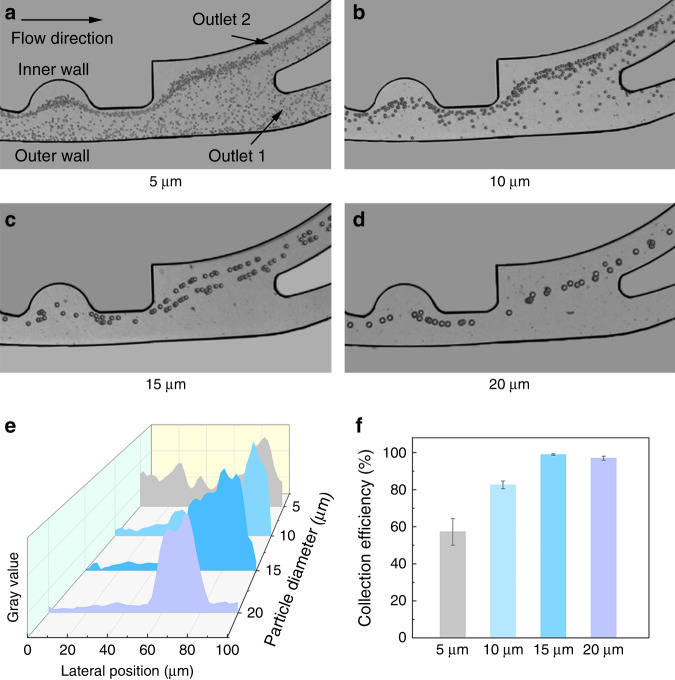
Particle experiment and analysis. **a** Composite image from experimental observation of 5 μm particles. **b** Composite image from experimental observation of 10 μm particles. **c** Composite image from experimental observation of 15 μm particles. **d** Composite image from experimental observation of 20 μm particles. **e** Lateral positions of different particles at the end of the CCEA channel. A narrower peak means a better focusing performance. **f** Collection efficiencies of different particles at outlet 2. The collection efficiencies of 10–20 μm particles were greater than 80%, which showed the capacity of the CCEA microfluidic device for particle washing.

Based on the flow rate ratio of 30: 90 μL/min, particles with four different diameters were tested in our CCEA microfluidic device. The initial numbers of 5, 10, 15, and 20 μm particles to be separated were 5 × 10^5^, 5 × 10^4^, 5 × 10^4^, and 5 × 10^4^, respectively. The volumes of all particle samples were 500 μL. The collection efficiency (the ratio of particles collected at outlet 2 to both outlets) was calculated to evaluate the focusing performance. All calculations were repeated more than three times and averaged. The competition between the inertial lift force *F*_L_ and the Dean drag force *F*_D_ determined the lateral migration of particles. When the particle diameter was 5 μm, as shown in Fig. 5a, a focusing position was generated close to the inner wall. However, according to calculations, the 5 μm particles collected at outlet 2 accounted for only 57.3% of the total. Such a small particle size led to poor focusing performance due to the weak *F*_L_. When the particle diameter was 10 μm, as shown in Fig. 5b, the particles were focused on a wide line close to the inner wall. Approximately 82.6% of the particles could be collected at outlet 2. Being more susceptible to the particle diameter (the *F*_L_ is proportional to the fourth power of the diameter of the particles, while the *F*_D_ is proportional to the diameter), the *F*_L_ contributed more to the lateral migration of the 10 μm particles. When the particle diameter was 15 μm, as shown in Fig. 5c, there were two focusing lines (an inner line and a less-inner line) entering outlet 2. The collection efficiency was nearly 100%. At this time, *F*_L_ and *F*_D_ were similar in magnitude. The particles on the inner focusing line were more affected by the *F*_D_, while the particles on the less-inner focusing line were more affected by the *F*_L_. When the diameter was 20 μm, as shown in Fig. 5d, the particles were focused in a line closer to the centerline. As the particle size increased, *F*_L_ exerted a greater effect on the particle migration. Fortunately, the *F*_D_ generated by our CCEA structure was still efficient enough to drive all particles to flow out from outlet 2. The lateral positions of different particles are shown in Fig. 5e. The narrow peaks of the 10–20 μm particles mean that they were focused in this CCEA structure. The collection efficiencies of different particles are shown in Fig. 5f.

**Fig. 6:**
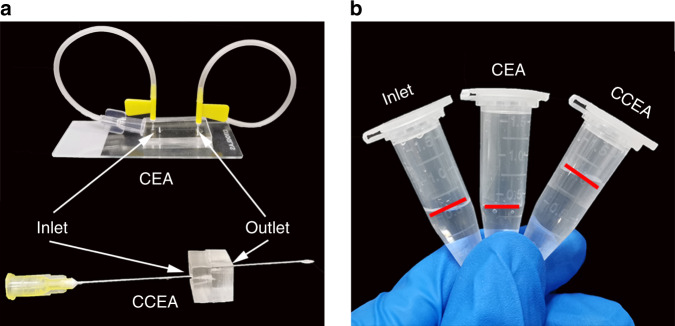
Comparison of sample consumption of the CEA device and the CCEA device. **a** Image of the CEA device and the CCEA device. **b** Volume comparison between the inlet, the outlet of the CEA device, and the outlet of the CCEA device. The particle loss rates were 64% and 7%, respectively.

In addition to the rapid focusing in such a short channel, another advantage of the CCEA device was low sample consumption. We conducted a comparative experiment between the CCEA device and the normal CEA device. As shown in Fig. 6a, the CEA device, like most microfluidic chips, was bonded with glass slides and needed two tubes for sample input and output. The radii of the tubes were 0.75 mm, and their lengths were 100 mm. The CCEA device was small enough to be directly connected with needles for sample input and output. The radius of the needle was 0.2 mm and its length was 28 mm. The size of both microchannels was small, so the particle loss could be ignored. The initial sample volume was 500 μL. The concentration of 15 μm particles in the sample was 1 × 10^5^/mL. For the normal CEA device, the amounts of fluid consumed in the input tube and the output tube were 225 and 190 μL, respectively. As shown in Fig. 6b, 360 μL fluid was collected at outlet 2. Under a flow rate ratio of 1:3 for the inlets and a flow rate ratio of 1:1 for the outlets, the particle concentration of the collection fluid was only half of the sample. Thus, the particle loss rate could be calculated to be 64%. For the CCEA device, the amounts of fluid consumed in the input tube and the output tube were 30 and 3.5 μL, respectively. After collection, 930 μL fluid was collected at outlet 2. The particle loss rate could be calculated to be 7%. If the target value of the collected particles was 5 × 10^4^, the time consumption values of the CEA device and the CCEA device were 28 and 17 min, respectively. These results showed that the needle-tip CCEA microfluidic device could collect target particles with low sample consumption by efficiently decreasing the particle loss rate.

### Cell washing

In Section 3.2, the fluid distribution showed that the sample solution can be collected at outlet 1. In Section 3.3, the target 10–20 μm particles were focused and collected at outlet 2. Thus, our CCEA microchannel showed the capability to remove extra solution (such as staining reagents) to realize cell washing. In addition, this device could remove insoluble wastes. Three human carcinoma cell lines, H1299 (average diameter ~19 μm), MCF-7 (average diameter ~17 μm), and U-2932 (average diameter ~11 μm), were mixed with 500 nm PS particles (representing waste that needs to be washed away) as samples. On the one hand, 500 nm particles were used to trace the sample fluid. Then, a more accurate washing efficiency could be calculated by flow cytometry. On the other hand, the 500 nm particles represented some insoluble wastes in the sample. For example, extracellular vesicles are small membrane vesicles of endocytic origin that are secreted by most cells in culture or in body fluids such as blood, urine, and saliva^46,47^. The size of exosomes ranges from 30 to 200 nm with an approximate median value of 100 nm^48^, whereas the size of some large EVs ranges from 200 to 1000 nm^49^. In a previous study on microfluidic devices, 100 and 500 nm particles were used to represent extracellular vesicles^50^. Thus, we chose 500 nm particles as waste in our research. To obtain a stable fluid distribution, a minimum flow rate ratio of 1:3 was needed. We noticed that the diameters of the common 3 mL syringe and 10 mL syringe were 8.18 and 14.72 mm, respectively. Under synchronous pushing, the flow rate ratio could be controlled at 1:3.2 using these two syringes. Thus, the washing process could be completed using only a double-channel syringe pump. On the same pump, the distance between these two syringes was so short that a common commercial infusion tube, instead of a custom tube, was feasible for connecting the sheath fluid and our CCEA microfluidic device. This can reduce equipment costs and simplify operations. Finally, the flow rates of the sample and sheath fluid were determined to be 25 and 80 μL/min, respectively.

**Fig. 7:**
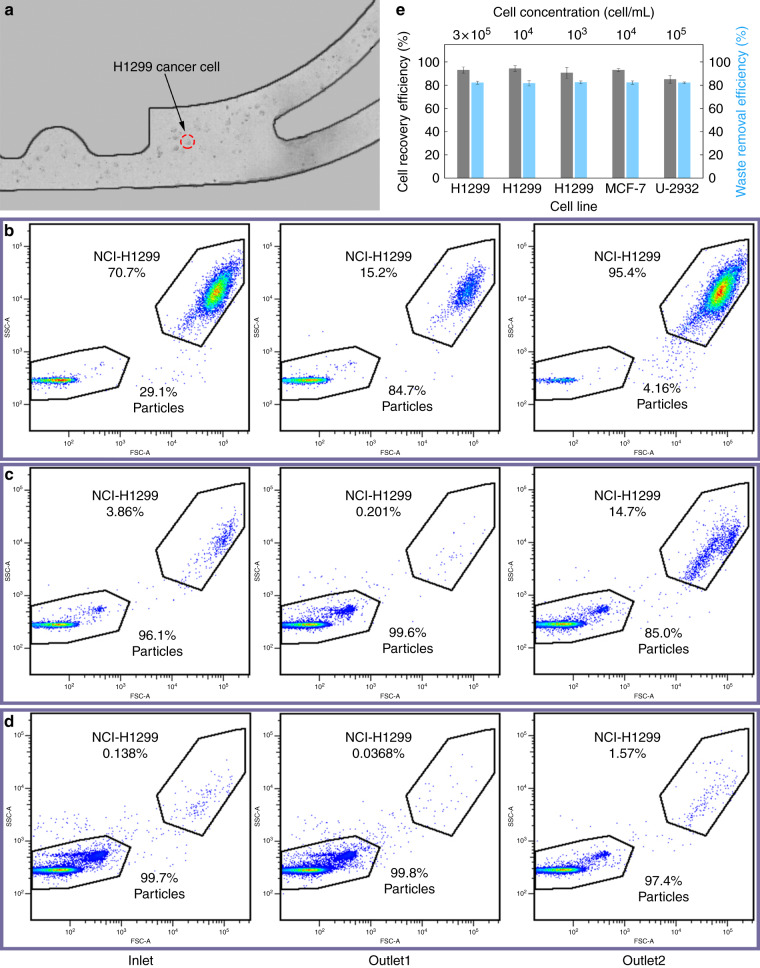
Cell experiments and analysis. **a** Composite image from experimental observation of cell washing. Sample and sheath fluid were injected from two syringes of different diameters. **b** Flow cytometry results when the concentration of H1299 cells was 3 × 10^5^/mL. **c** Flow cytometry results when the concentration of H1299 cells was 1 × 10^4^/mL. **d** Flow cytometry results when the concentration of H1299 cells was 1 × 10^3^/mL. **e** The waste removal efficiencies and the cell collection efficiencies at different cell concentrations and different cell lines.

**Fig. 8:**
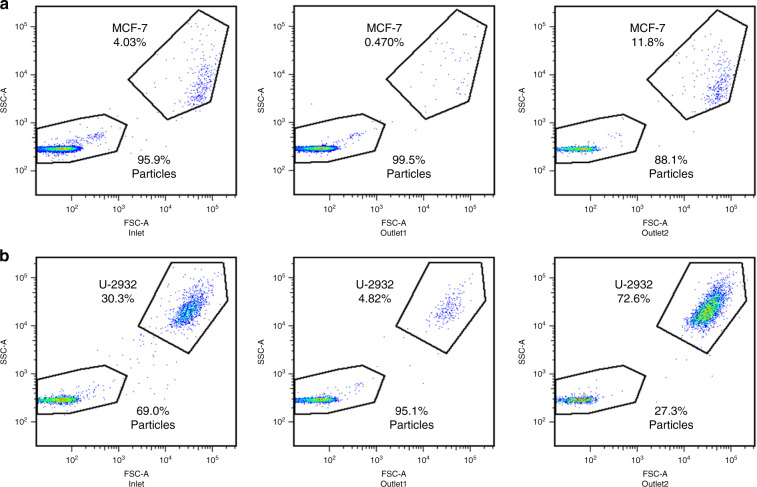
Flow cytometry results of MCF-7 cells and U-2932 cells. **a** The concentration of MCF-7 cells was 1 × 10^4^/mL. **b** The concentration of U-2932 cells was 1 × 10^5^/mL.

The migration of H1299 cells was observed in brightfield, and the cell collection efficiency after washing was calculated based on a flow cytometer. The volumes of all cell samples were 500 μL. To obtain a cell trajectory in brightfield, the concentration of H1299 was first set to 3 × 10^5^/mL. As shown in Fig. 7a, most cells could migrate toward the inner wall and be collected at outlet 2. However, they could no longer be focused in a line due to their nonuniform cell size. The flow cytometry results shown in Fig. 7b demonstrate the proportions of cells collected at the inlet, outlet 1, and outlet 2. In the plots of the forward scatter (FSC) and side scatter (SSC), H1299 cells and PS particles could be distinguished easily due to their large size difference. In the initial sample, the ratios of H1299 cells and PS particles to the total number of cells were 70.7 and 29.1%, respectively. After the washing, this ratio for H1299 cells increased to 95.4% in the outlet 2 collection, while it increased to 84.7% for the PS particles in the outlet 1 collection. To model rare cells, the H1299 cell concentrations were further set to 1 × 10^4^/mL and 1 × 10^3^/mL. Fluorescent signals were used to ensure gate accuracy at such low concentrations. The flow cytometry results are shown in Fig. 7c,d. The cell collection efficiency of H1299 cells ranged from 91.9 ± 2.6 to 94.3 ± 2.5% and 90.6 ± 4.7% as the cell concentrations decreased from 3 × 10^5^/mL to 1 × 10^4^/mL and 1 × 10^3^/mL (Fig. 7e). In addition, as shown in Fig. 8, the adherent human breast cancer cell line MCF-7 (cell collection efficiency 93.0 ± 1.4%) and suspension human diffuse large B lymphoma cell line U-2932 (cell collection efficiency 85.0 ± 3.3%) were tested. The sample volumes were both 500 μL, and the cell concentrations were 1 × 10^4^/mL and 1 × 10^5^/mL. The waste removal efficiencies in all cases were ~81–84%. The high cell collection efficiency and stable waste removal efficiency reflected the universality of the CCEA microfluidic device for cell washing. We also tested 1 μm particles as wastes. However, many 1 μm particles migrated toward the inner wall and flowed out from outlet 2. Therefore, the critical size for the particle that can be washed in this device was 500 nm.

**Fig. 9:**
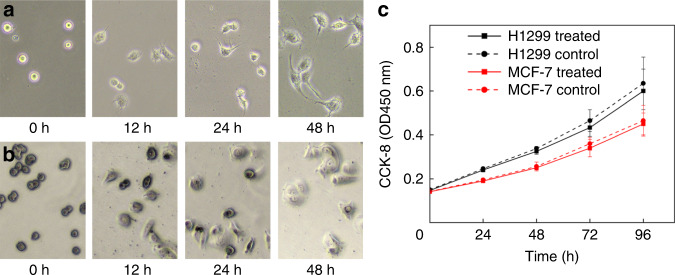
Biocompatibility of the CCEA device. **a** Images of the H1299 cell morphology after washing using a CCEA microfluidic device. **b** Images of the MCF-7 cell morphology after washing using a CCEA microfluidic device. **c** Cell proliferation of H1299 cells and MCF-7 cells.

The cell viabilities and morphologies of H1299 cells and MCF-7 cells after washing were investigated. As shown in Fig. 9a, b, both H1299 cells and MCF-7 cells displayed normal adherent growth and proliferation after 48 h. The proliferation rates of the collected cells and untreated cells were measured by CCK-8. The cell suspensions were inoculated in 96-well plates. CCK-8 solution was added to the wells at 24, 48, 72, and 96 h. The plate was incubated for 1–4 h in an incubator. The absorbance was measured at 450 nm using a microplate reader. As shown in Fig. 9c, both the collected cells and untreated cells proliferated normally. The cell activity can be calculated as:

4 $${\mathrm{Cell}}\,{\mathrm{activity}} \times 100\% = \frac{{A_r - A_b}}{{A_c - A_b}} \times 100\%$$

where *A*_r_ is the absorbance of a treated well that contains collected cells, medium, and CCK-8 solution; *A*_c_ is the absorbance of control well that contains untreated cells, medium, and CCK-8 solution; and *A*_b_ is the absorbance of a blank well that only contains medium and CCK-8 solution. The cell activities of H1299 cells and MCF-7 cells were calculated to be 93.8 and 97.5%, respectively. This high cell activity showed the negligible cell damage and high biocompatibility of the CCEA microfluidic device. The diameters of the cells used in our experiments were much smaller than the channel size. At low cell concentrations, monodispersed cells could smoothly flow through the whole channel. In general, blockages rarely occurred, and the performance of the device was stable.

## Conclusions

We demonstrated a CCEA microchannel that realizes continuous, rapid, and sample-saving cell washing on a needle tip. Compared with the circle channel and CEA channel, our CCEA microchannel can produce a much stronger Dean flow. Under the enhanced Dean drag force, target particles rapidly migrated from the initial outer wall (pinched by sheath fluid) to the inner wall side. The same particles were dominated by the inertial lift force and focused at the centerline in the circle channel and CEA channel. To obtain a stable fluid distribution, the critical flow rate ratio and the critical total flow rate were determined to be 1:3 and 120 μL/min, respectively. Both a higher flow rate and a higher flow rate ratio may cause the initial sample layer to disperse into outlet 2 (for cell collection). For rapid particle/cell focusing, 57.3% of 5 μm particles, 82.6% of 10 μm particles, and nearly 100% of 15/20 particles could be transferred from the sample layer and collected at outlet 2. When 500 μL of a rare sample were treated, the particle loss rate was only 7%, whereas it was 64% in the normal CEA channel. Under an optimal total flow rate of 120 μL/min, cell washing could be completed in 17 min. For cell experiments, H1299 cells were washed from 500 nm particles. The flow rate ratio was controlled by the diameters of different syringes and determined to be 25 (sample) and 80 μL/min (sheath fluid). The cell collection efficiency of H1299 cells ranged from 91.9 ± 2.6 to 94.3 ± 2.5% and 90.6 ± 4.7% as the cell concentrations decreased from 3 × 10^5^/mL to 1 × 10^4^/mL and 1 × 10^3^/mL, respectively. The cell collection efficiencies of MCF-7 cells and U-2932 cells were 93.0 ± 1.4 and 85.0 ± 3.3%, respectively. The waste removal efficiencies of 500 nm particles were ~81–84%. In conclusion, this extremely small-sized CCEA microchannel can efficiently wash cells from rare samples on a needle tip, showing potential for biological applications.

## References

[1] Oomen PE, Aref MA, Kaya I, Phan NTN, Ewing AG (2018). Chemical analysis of single cells. Anal. Chem..

[2] Kieninger J, Weltin A, Flamm H, Urban GA (2018). Microsensor systems for cell metabolism – from 2d culture to organ-on-chip. Lab Chip.

[3] Hachey SJ, Hughes CCW (2018). Applications of tumor chip technology. Lab Chip.

[4] Mohamed TMA (2018). Regulation of cell cycle to stimulate adult cardiomyocyte proliferation and cardiac regeneration. Cell.

[5] Movellan J (2014). Amphiphilic dendritic derivatives as nanocarriers for the targeted delivery of antimalarial drugs. Biomaterials.

[6] Tucker AT (2018). Discovery of next-generation antimicrobials through bacterial self-screening of surface-displayed peptide libraries. Cell.

[7] Molina-Miras A (2018). Long-term culture of the marine dinoflagellate microalga Amphidinium carterae in an indoor LED-lighted raceway photobioreactor: production of carotenoids and fatty acids. Bioresour. Technol..

[8] Shekhawat LK, Sarkar J, Gupta R, Hadpe S, Rathore AS (2018). Application of CFD in bioprocessing: separation of mammalian cells using disc stack centrifuge during production of biotherapeutics. J. Biotechnol..

[9] Molina-Miras A (2019). A new approach to finding optimal centrifugation conditions for shearsensitive microalgae. Algal Res..

[10] Peterson BW, Sharma PK, van der Mei HC, Busscher HJ (2012). Bacterial cell surface damage due to centrifugal compaction. Appl. Environ. Microbiol..

[11] Mata C (2009). Cell motion and recovery in a two-stream microfluidic device. Microfluidics Nanofluidics.

[12] Perotti CG (2004). A new automated cell washer device for thawed cord blood units. Transfusion.

[13] Tarn MD, Lopez-Martinez MJ, Pamme N (2014). On-chip processing of particles and cells via multilaminar flow streams. Anal. Bioanal. Chem..

[14] Doonan SR, Lin M, Bailey RC (2019). Droplet CAR-Wash: continuous picoliter-scale immunocapture and washing. Lab Chip.

[15] Huang Y (2020). Isolation of circulating fetal trophoblasts by a four-stage inertial microfluidic device for single-cell analysis and noninvasive prenatal testing. Lab Chip.

[16] Lee D, Choi Y, Lee W (2020). Enhancement of inflection point focusing and rare-cell separations from untreated whole blood. Lab Chip.

[17] Gossett DR (2012). Inertial manipulation and transfer of microparticles across laminar fluid streams. Small.

[18] Zhou J, Papautsky I (2019). Size-dependent enrichment of leukocytes from undiluted whole blood using shear-induced diffusion. Lab Chip.

[19] Amini H, Lee W, Di Carlo D (2014). Inertial microfluidic physics. Lab Chip.

[20] Sun J (2012). Double spiral microchannel for label-free tumor cell separation and enrichment. Lab Chip.

[21] Hou HW, Bhattacharyya RP, Hung DT, Han J (2015). Direct detection and drug-resistance profiling of bacteremias using inertial microfluidics. Lab Chip.

[22] Yin L (2018). Microfluidic label-free selection of mesenchymal stem cell subpopulation during culture expansion extends the chondrogenic potential in vitro. Lab Chip.

[23] Johnston ID (2014). Dean flow focusing and separation of small microspheres within a narrow size range. Microfluidics Nanofluidics.

[24] Bhagat AAS (2011). Pinched flow coupled shear-modulated inertial microfluidics for high-throughput rare blood cell separation. Lab Chip.

[25] Zhang J (2016). Fundamentals and applications of inertial microfluidics: a review. Lab Chip.

[26] Wu Z, Chen Y, Wang M, Chung AJ (2016). Continuous inertial microparticle and blood cell separation in straight channels with local microstructures. Lab Chip.

[27] Warkiani ME (2015). Malaria detection using inertial microfluidics. Lab Chip.

[28] Park J, Jung H (2009). Multiorifice flow fractionation: continuous size-based separation of microspheres using a series of contraction/expansion microchannels. Anal. Chem..

[29] Cha S (2014). Hoop stress-assisted three-dimensional particle focusing under viscoelastic flow. Rheologica Acta.

[30] Lee MG (2013). Label-free cancer cell separation from human whole blood using inertial microfluidics at low shear stress. Anal. Chem..

[31] Shen S (2017). Spiral microchannel with ordered micro-obstacles for continuous and highly-efficient particle separation. Lab Chip.

[32] Gou Y (2020). Sheathless inertial focusing chip combining a spiral channel with periodic expansion structures for efficient and stable particle sorting. Anal. Chem..

[33] Xiang N, Dai Q, Han Y, Ni Z (2019). Circular-channel particle focuser utilizing viscoelastic focusing. Microfluidics Nanofluidics.

[34] Yun SS (2010). Handheld mechanical cell lysis chip with ultra-sharp silicon nano-blade arrays for rapid intracellular protein extraction. Lab Chip.

[35] Song S, Kim MS, Lee J, Choi S (2015). A continuous-flow microfluidic syringe filter for size-based cell sorting. Lab Chip.

[36] Nunes Pauli GE (2015). Lab-in-a-syringe using gold nanoparticles for rapid immunosensing of protein biomarkers. Lab Chip.

[37] Xiang N (2019). Flow stabilizer on a syringe tip for hand-powered microfluidic sample injection. Lab Chip.

[38] Xu H, Liao C, Zuo P, Liu Z, Ye BC (2018). Magnetic-based microfluidic device for on-chip isolation and detection of tumor-derived exosomes. Anal. Chem..

[39] Qian C (2021). Rapid exosomes concentration and in situ detection of exosomal microRNA on agarose-based microfluidic chip. Sens. Actuators B: Chem..

[40] Zhao Z, Yang Y, Zeng Y, He M (2016). A microfluidic ExoSearch chip for multiplexed exosome detection towards blood-based ovarian cancer diagnosis. Lab Chip.

[41] Vaidyanathan R (2014). Detecting exosomes specifically: a multiplexed device based on alternating current electrohydrodynamic induced nanoshearing. Anal. Chem..

[42] Mcdonald JC, Whitesides GM (2010). ChemInform abstract: poly(dimethylsiloxane) as a material for fabricating microfluidic devices. ChemInform.

[43] Nielsen JB (2019). Microfluidics: innovations in materials and their fabrication and functionalization. Anal. Chem..

[44] Di Carlo D (2009). Particle segregation and dynamics in confined flows. Phys. Rev. Lett..

[45] Yuan D (2015). Dean-flow-coupled elasto-inertial three-dimensional particle focusing under viscoelastic flow in a straight channel with asymmetrical expansion–contraction cavity arrays. Biomicrofluidics.

[46] Thery C, Zitvogel L, Amigorena S (2002). Exosomes: composition, biogenesis and function. Nat. Rev. Immunol..

[47] Raposo G, Stoorvogel W (2013). Extracellular vesicles: exosomes, microvesicles, and friends. J. Cell Biol..

[48] Melo SA (2015). Glypican-1 identifies cancer exosomes and detects early pancreatic cancer. Nature.

[49] Hannafon BN, Ding WQ (2013). Intercellular communication by exosome-derived microRNAs in cancer. Int. J. Mo. l Sci..

[50] Liu C (2017). Field-free isolation of exosomes from extracellular vesicles by microfluidic viscoelastic flows. ACS Nano.

[51] Kuntaegowdanahalli SS, Bhagat AAS, Kumar G, Papautsky I (2009). Inertial microfluidics for continuous particle separation in spiral microchannels. Lab Chip.

[52] Zhang J, Li M, Li WH, Alici G (2013). Inertial focusing in a straight channel with asymmetrical expansion–contraction cavity arrays using two secondary flows. J. Micromech. Microeng..

